# Testing the construct validity of a health transition question using vignette-guided patient ratings of health

**DOI:** 10.1186/s12955-017-0832-4

**Published:** 2018-01-03

**Authors:** Michael M. Ward, Jinxiang Hu, Lori C. Guthrie, Maria Alba

**Affiliations:** Intramural Research Program, National Institute of Arthritis and Musculoskeletal and Skin Diseases, National Institutes of Health, Building 10 CRC, Room 4-1339, 10 Center Drive, Bethesda, MD 20892 USA

**Keywords:** Transition question, Anchors, Vignettes, Self-rated health

## Abstract

**Background:**

A single-item transition question is often used to assess improvement or worsening in health, but its validity has not been tested extensively. The purpose of this study was to test the construct validity of a transition question by relating it to qualitative changes in patient’s self-rating of health guided by clinical vignettes.

**Methods:**

We studied 169 patients with active rheumatoid arthritis (RA) before and after treatment escalation. At both assessments, patients scored their current health on a rating scale after first rating three vignettes describing mild, moderate, or severe RA. We classified patients into one of these three RA categories using a nearest-neighbor match. We then related the change in these self-rated categories between visits to responses to a transition question on visit 2.

**Results:**

Sixty patients improved their RA vignette category after treatment, 86 remained in the same vignette category, and 23 worsened categories. On the transition question, 101 patients reported improvement, 48 reported no change, and 20 reported worsening, representing a modest association with changes in RA vignette categories (polychoric correlation *r* = 0.19). The association was stronger if patients who were in the mild RA category at both visits were also classified as improved if their self-rating changed from below to above their mild vignette rating (*r* = 0.23) and when incorporating the importance of changes on the transition question (*r* = 0.26).

**Conclusion:**

Changes in health states, guided by clinical vignettes, support the construct validity of the transition question.

## Background

Transition questions, also known as global ratings of change, are judgments by patients of whether their health has improved, worsened, or is unchanged from a prior time or from the start of an intervention [1]. Given its simplicity and face validity, the transition question is among the most commonly used measures in clinical practice and clinical investigations. In particular, transition questions are frequently used to validate other measures, and serve as anchors for estimating the minimal clinically important changes in other health status measures [2, 3].

Questions have arisen about the validity of transition questions [4]. The cognitive processes that patients use to judge whether a qualitative change in health has occurred are complex and incompletely understood [5, 6]. Judgments of change have been shown to be influenced not only by the measured change in health but also by how well or poorly the patient feels when making the judgment [7–10]. The post-intervention state of health has often been found to be more influential than the pre-intervention state of health, which violates the premise that transition questions should be reciprocally associated with both the pre- and post-intervention states of health [11]. Countering these concerns is evidence that despite some contribution from the post-intervention state, transition questions are strongly associated with measured changes in health, and responses to transition questions parallel associations with other anchors, such as physician ratings of improvement [12–14].

In a new approach to test the construct validity of transition questions, we used clinical vignettes as indicators of changes in patient’s health. Vignettes have been increasingly used in health status measurement because they may provide less biased ratings than other patient-reported measures, or at a minimum can identify the extent of intergroup bias [15–18]. We asked patients to rate three clinical vignettes that described different levels of health (mildly ill, moderately ill, and severely ill), and matched patients’ ratings of their current health to one of these three levels before and after a medication intervention. Using the monotrait-heteromethod framework for convergent validity, we considered that patients who experienced a major subjective change in health with treatment would endorse this change on both their vignette rating and the transition question, while those with no change in health based on their vignette ratings would not report a change on the transition question [19].

## Methods

### Subjects and study design

Data were from a short-term prospective longitudinal study whose main goal was to determine estimates of minimal clinically important improvement for measures of rheumatoid arthritis (RA) activity [20]. We enrolled adults with active RA from two outpatient clinics who, in the judgment of their rheumatologist, required treatment escalation with either disease-modifying medications, biologicals, or prednisone. Subjects were evaluated twice, once at study entry and then either 1 month later (for those treated with prednisone) or 4 months later (all others). The timing of the follow-up visit differed by treatment group because clinical responses occur more rapidly with prednisone treatment. Subjects had a joint examination at each visit, and completed self-reported measures of health status and quality of life at both visits. We used a global transition question as the anchor for determining thresholds for clinically important improvement in RA activity measures [21]. The study protocol was approved by the institutional review board, and all subjects provided written informed consent.

Of 262 subjects enrolled, 206 subjects participated in a substudy which included the rating of clinical vignettes. Lack of time and primary language other than English were the main reasons for non-participation in this substudy. Of these, 195 subjects completed the vignette ratings at both visits. We then excluded 26 subjects who rated the mild RA vignette as less healthy than the severe RA vignette, which indicated some confusion with the task. This resulted in 169 subjects for analysis (82% of substudy participants).

### Clinical vignette ratings

We used a computer-administered questionnaire to obtain subjects’ ratings of their own current health and ratings of three hypothetical health states described in vignettes. Responses were recorded on a vertical visual analog scale with the top endpoint labeled “perfect health” and the bottom endpoint labeled “worst imaginable health,” which were set to 1.0 and 0, respectively. The scale was unnumbered. Subjects were asked to move a cursor with the mouse to the point on the scale that corresponded to their rating on the perfect-worst continuum, which was then recorded by the computer. First, subjects read a description of a person with mild RA and then rated this vignette. This was followed by descriptions and ratings of a person with moderate RA and then severe RA, in this order. Subjects were then asked to rate their current health on this scale, considering the endpoints and their vignette ratings [22]. Specifically, the prompt was “Now think about how your arthritis affects you currently. Think about your ability to work and do things, your ability to take care of yourself, your ability to enjoy leisure activities, the amount of pain you have, your mood, and your outlook for the future.”

The three clinical vignettes were based on the McMaster Utility Measurement Questionnaire [23]. The mild RA vignette read as follows: Think what it would be like to live in the following way: You are able to perform all of your daily activities, like work, shopping and driving. You are completely able to take care of your personal needs, like eating and bathing. You have some difficulty participating in leisure activities, like sports and hobbies. You have occasional pain. You normally do not have any worry or stress, but sometimes you are concerned about the future course of your arthritis. You have some mild stomach upset from some medication you take.

The moderate RA vignette was: Think what it would be like to live in the following way: On most days you are able to run errands and work around the house, but fatigue and joint pain prevent you from working. You are completely able to take care of your personal needs, such as eating and bathing. Joint pain is mild on most days, but is never gone and is sometimes quite severe. You rarely have enough energy for leisure activities. At times you are frustrated with dealing with your arthritis. The medication you take sometimes causes diarrhea.

The severe RA vignette read as follows: Think what it would be like to live in the following way: You are unable to work, shop, or drive. You have much difficulty getting around outside the house. Sometimes you need help to bathe. You are unable to participate in any leisure activities. You are depressed and frustrated. You have severe pain on most days. The medications you take cause you painful sores in your mouth and difficulty thinking.

Subjects performed new ratings at the second visit, without knowledge of their prior ratings.

### Transition question

Since the vignettes portrayed overall RA status, we used the global transition question for this analysis. At the second visit, subjects were asked to respond to the following on a written questionnaire: “Since the start of the study, overall my arthritis has: improved, stayed the same, or worsened.” Those who responded that they were improved or worsened were then asked to rate the importance of the change on a seven-level scale: almost none, hardly at all; a little important; somewhat important; moderately important; a good deal important; very important; extremely important [24].

### Statistical analysis

We first categorized patient’s self-ratings on the visual analog scale into one of three states, based on the proximity of their self-rating to their ratings of the mild, moderate, or severe RA vignette, using the nearest neighbor technique [25]. For example, consider a subject whose mild, moderate and severe vignette ratings were 0.88, 0.65, and 0.34, respectively. If their self-rating was 0.72, they would be classified as moderate, whereas if their self-rating was 0.10, they would be classified as severe. Classification was done independently for each visit. Subjects were only classified with respect to their personal ratings of the vignettes and not to group mean vignette ratings. In the rare event of a nearest-neighbor tie, we randomly assigned the subject to one of the categories.

We then compared each subject’s category at the first visit to that at the second visit. In the base analysis, those who moved from severe to either moderate or mild, or from moderate to mild, were classified as improved. Those who moved from mild to either moderate or severe, or from moderate to severe, were classified as worsened. These shifts represented subjects who no longer rated themselves as similar to one vignette and more like the person described in a different vignette, which we interpreted as a clinically meaningful change in the subject’s perception of their health status related to RA. The remainder were classified as unchanged. We then related changes in subjects’ health categories, guided by the clinical vignettes, to their responses on the transition question (improved, same, or worsened) using polychoric correlations. Polychoric correlations test the association between two ordinal variables, with the assumption that the data underlying the ordered categories are continuous latent traits. Model fit was tested using the likelihood ratio chi-square test G^2^.

We repeated the analysis using subjects’ ratings of the importance of changes on the transition question, creating a 15-point scale ranging from −7 for those who reported worsening that was extremely important to +7 for those who reported improvement that was extremely important, with 0 representing those who reported no change.

In the base analysis, subjects who were classified as mild on the first visit had no opportunity to register improvement, which may have limited the associations with the transition question. Therefore, we repeated the analysis with the modification that those subjects who were categorized as mild at both visits, but whose self-rating at visit 1 was less healthy than their rating of the mild vignette and whose self-rating at visit 2 was healthier than their mild vignette rating, were also classified as improved.

To our knowledge, there are no accepted criteria for interpreting the magnitude of associations based on polychoric correlations. Therefore, we adopted the convention used for point-biserial correlations (i.e. between one ordinal variable and one dichotomous variable). Following Cohen, medium effect sizes are represented by point-biserial correlations of 0.243, which correspond to Cohen’s d of 0.5 [26, 27]. We considered polychoric correlations between the vignette-guided self-ratings and the transition question responses of 0.243, representing a medium effect, to support the construct validity of the transition question.

## Results

### Subject characteristics

The study included 124 women (73%) and 45 men, with a mean (± standard deviation) age of 53.2 ± 13.7 years and a median (25th, 75th percentile) duration of RA of 6.5 (2.3, 16.0) years. Subjects had active RA at entry, with a mean Disease Activity Score-28 (a composite score of tender and swollen joint counts and serum C-reactive protein level; possible range 1–9.4 [20]) of 5.3 ± 1.0. At visit 2, the mean with-subject Disease Activity Score-28 change was −0.9 points, indicating improvement on the group level.

### Clinical vignette ratings

Mean vignette ratings at each visit are shown in Table 1. There were no significant intra-subject differences in ratings of the mild vignette or moderate vignette between the two visits, while the severe vignette was rated as slightly higher on the second visit. In comparison, subjects’ self-ratings improved substantially. We did not observe any distress or annoyance among subjects during the vignette ratings.

**Table 1:** Clinical vignette ratings and self ratings at each study visit. Values are mean ± standard deviation

	Visit 1	Visit 2	Within-subject difference	P^a^
Mild vignette	0.73 ± 0.18	0.76 ± 0.16	0.02 ± 0.19	0.10
Moderate vignette	0.51 ± 0.19	0.53 ± 0.19	0.03 ± 0.24	0.16
Severe vignette	0.20 ± 0.14	0.24 ± 0.19	0.04 ± 0.20	0.03
Self rating	0.55 ± 0.25	0.68 ± 0.21	0.13 ± 0.22	< 0.0001

^a^based on paired t test

Based on the nearest neighbor approach, 36 subjects had self-ratings that were closest to the severe vignette at visit 1, while 51 subjects were classified as moderate and 82 as mild (Table 2). At the second visit, 60 subjects had improved by at least one category using the nearest neighbor, but 23 worsened by at least one category. Self-ratings increased by an average of 0.28 ± 0.21 among those who improved by at least one category, were unchanged among those who remained in the same vignette category (0.09 ± 0.17), and decreased an average of −0.11 ± 0.16 among those whose vignette category worsened.

**Table 2:** Classification of subject’s rheumatoid arthritis status based on their self-ratings relative to their ratings of the clinical vignettes at each visit

		Visit 2			
		Severe	Moderate	Mild	Total
Visit 1	Severe	8	15	13	36
	Moderate	4	15	32	51
	Mild	4	15	63	82
	Total	16	45	108	169

### Association of vignette-guided self-ratings with transition question responses

In the base analysis, 101 subjects reported improvement on the transition question, 48 subjects reported no change, and 20 subjects reported worsening (Table 3). These responses were only modestly associated with changes in categories of RA status based on self-ratings guided by the vignettes, with *r* = 0.19. Model fit was good (G^2^ = 0.29; *p* = 0.96). In the analysis that accommodated improvement among subjects in the mild vignette category at visit 1, the association was somewhat stronger (*r* = 0.23), approaching the criterion of a medium effect (Table 3). This model also fit the data well (G^2^ = 0.53; *p* = 0.91).

**Table 3:** Association between the transition question and improvement or worsening in category of rheumatoid arthritis status based on self-ratings guided by clinical vignettes

		Base analysis				Improvement allowed in those with mild self-rating at visit 1			
		*r* = 0.19				*r* = 0.23			
		Self-ratings guided by vignettes				Self-ratings guided by vignettes			
		Worsened	Unchanged	Improved	Total	Worsened	Unchanged	Improved	Total
Transition Question	Worsened	5	10	5	20	5	10	5	20
	Stayed the same	7	26	15	48	7	21	20	48
	Improved	11	50	40	101	11	39	51	101
	Total	23	86	60	169	23	70	76	169

### Association of vignette-guided self-ratings with importance of the transition question changes

Not all of the 15 possible importance ratings were used by subjects, resulting in 12 categories for the importance of the change in the transition question (Table 4). Vignette-guided self-ratings were more strongly associated with the importance ratings of the transition question than with the 3-category transition question. In the base analysis, *r* = 0.26 (model fit G^2^ = 24.3; *p* = 0.33), while in the analysis that accommodated improvement among subjects in the mild category at visit 1, *r* = 0.28 (model fit G^2^ = 15.8; *p* = 0.78). Both associations represent a medium effect, supporting the construct validity of the transition question.

**Table 4:** Association between the subjects’ ratings of the importance of change on transition question and improvement or worsening in category of rheumatoid arthritis status based on self-ratings guided by clinical vignettes

		Base analysis				Improvement allowed in those with mild self-rating at visit 1			
		*r* = 0.26				*r* = 0.28			
		Self-ratings guided by vignettes				Self-ratings guided by vignettes			
		Worsened	Unchanged	Improved	Total	Worsened	Unchanged	Improved	Total
Transition Question	Extremely important worsening (−7)	2	4	1	7	2	4	1	7
	Very important worsening (−6)	1	1	1	3	1	1	1	3
	Good deal important worsening (−5)	2	3	1	6	2	3	1	6
	Little important worsening (−2)	0	2	1	3	0	2	1	3
	Hardly important at all worsening (−1)	0	0	1	1	0	0	1	1
	No change (0)	7	26	15	48	7	21	20	48
	Little important improvement (+2)	1	2	1	4	1	2	1	4
	Somewhat important improvement (+3)	0	2	1	3	0	1	2	3
	Moderately important improvement (+4)	1	0	2	3	1	0	2	3
	Good deal important improvement (+5)	5	10	4	19	5	6	8	19
	Very important improvement (+6)	2	14	9	25	2	13	10	25
	Extremely important improvement (+7)	2	22	23	47	2	17	28	47
	Total	23	86	60	169	23	70	76	169

## Discussion

The purpose of a transition question is to capture a patient’s explicit judgment about whether they have experienced a noticeable change in their health. One method to test the validity of these explicit judgments is to compare how well they relate to contemporaneous implicit judgements of important change. We used shifts in subjects’ ratings of their health relative to a set of three health states described in clinical vignettes as an indicator of a change in their implicit judgments. We found that changes in vignette-guided categories of health after treatment escalation were associated with similar changes in the transition question, with associations of medium effect sizes. These results support the construct validity of the global transition question.

Several previous studies that reported only weak or no associations between changes in health status measures and a transition question examined patients in usual care settings who were not necessarily very symptomatic at study entry or who did not receive specific or impactful treatment during the study [1, 4, 8–10]. Some studies did not report the magnitude of health changes observed [1, 7–9]. These naturalistic designs might have limited the ability of these studies to detect associations with the transition question, because few patients may have had notable changes in health. Other studies used health status measures of limited responsiveness [28]. Studies that examined major categories of transition responses (i.e. improved, same, or worsened) were more likely to find associations with changes in health status measures than studies that used more finely-graded responses or that converted transition question responses to a semi-quantitative score based on the perceived importance of the change [12, 29, 30]. These semi-quantitative measures include two potential sources of error or imprecision: one related to the direction of change in the transition question and one related to the importance of that change. Associations with qualitative changes in the transition question may be obscured by imprecision in the importance ratings.

In contrast, we examined a group of patients with active disease who were all treated with known effective medications. The importance of studying patients with active disease is underscored by a study of highly active antiretroviral therapy that found associations between health measures and a transition question among patients with symptomatic human immunodeficiency virus disease but not among patients who were asymptomatic [14]. The magnitude of associations with the transition question was stronger when ratings of the importance of the change were analyzed than when examining only the presence and direction of a change. These results suggest that information contributed by the subjective importance of the change may outweigh any associated measurement error.

Our study has some limitations. We chose the reference standard to which the transition question was compared to be a substantial and clinically meaningful change in health, denoted by a change in self-rating relative to the vignettes. We chose this approach, rather than using the rating scale response as a continuous measure, in order to emphasize major changes rather than incremental changes in health, even though mapping patients to the vignettes might have encumbered some error. We do not know if subjects would have picked the same states to describe themselves if asked to do so explicitly. However, asking subjects to directly compare themselves to a vignette has been shown to induce agreement irrespective of the health state described in the vignette, increasing the likelihood of disordered responses compared to a sequential rating of vignettes followed by a self-rating [22]. We could have included more vignettes, although with finer categories, the distinctions between health states would decrease and we might be less certain that a change in health state represented an important change. Random errors and disordered ratings also increase with the number of vignettes. Three vignettes have commonly been used in health studies, although one study used 10 vignettes [16, 17, 31, 32]. Thirteen percent of potential subjects were excluded because of disordered vignette ratings, which is similar to the frequency reported in other surveys [33]. One reason for disordered responses may be that subjects keyed in on one specific feature of a particular vignette description. It is not clear if the vignettes served to improve recall of the subject’s prior health state, which is a well-recognized bias affecting transition questions [5, 6]. In this study, we used the vignettes, which required more time and respondent burden, to validate the simple single-item transition question.

Use of clinical vignettes assumes response consistency, that is, that subjects use the same criteria and standards to rate themselves as they use when rating the vignettes. Few studies have tried to test this assumption, with variable results [34–36]. Response consistency may be enhanced by using multidimensional vignettes rather than vignettes focused on a single health domain, and by reminding subjects to identify with the person described in the vignette [36]. We employed both aspects in this study. A second major assumption is vignette equivalence, wherein all respondents view the vignettes as describing the same construct with the same unidimensional scale. Vignette equivalence is central to their use in estimating differential item functioning among groups of patients, but is less relevant in our study because vignette ratings were not compared across subjects. We did not explore potential differences among patient subsets in the absence of evidence to support effect modification. Vignettes may also be a novel method to assess response shift, particularly recalibration [15]. Our finding of small intra-subject differences in vignette ratings over time suggest the absence of major response shift.

## Conclusions

Our results support the construct validity of a general health transition question. Replication in other conditions and settings would be important, with attention to the key design features of studying patients with active symptoms who experienced major changes in health.

## Abbreviations

RA: Rheumatoid arthritis
